# Transgender Day of Visibility 2022: an interview with Adam Armada-Moreira and Ave Bisesi on trans experiences in STEM

**DOI:** 10.1038/s42003-022-03247-6

**Published:** 2022-03-31

## Abstract

This year at *Communications Biology*, we wanted to celebrate Transgender Day of Visibility by highlighting researchers at multiple career stages. In this Q&A, we asked early-career biologists about their own achievements, academic experiences, and how STEM can better support trans researchers.

Dr. Adam Armada-Moreira (he/him) is a postdoctoral fellow at Linköping University in Sweden, where he studies plant electrophysiology and organic bioelectronics.

**Figure:**
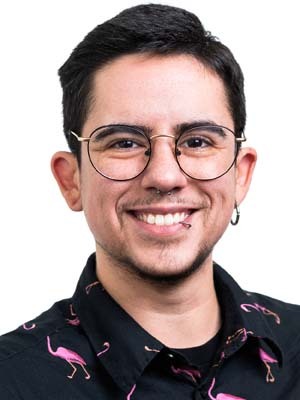
Adam Armada-Moreira

Ave Bisesi (they/them/he/him) is a second-year PhD student in the Ecology, Evolution and Behavior program at the University of Minnesota, where they study the evolutionary ecology of microbial communities and impact of bacteriophages on bacterial interactions.

**Figure:**
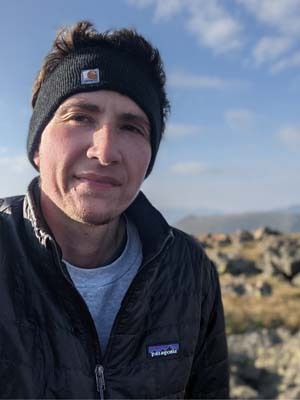
Ave Bisesi

Please tell us about your academic background and current research interests.

**Adam Armada-Moreira (AAM):** My academic path has been a very interesting and somewhat surprising journey. Out of high school, I just knew I loved science and wanted to study it, so I went on for a bachelor’s degree in Biochemistry at the Faculty of Sciences of the University of Lisbon, Portugal. This made me fall head over heels for biological sciences and led me to my next step with a master’s degree in Neuroscience, at the Faculty of Medicine of the University of Lisbon, with my thesis project focusing on oligodendrogenesis; the differentiation of oligodendrocytes induced by pharmacological treatments.

I then started my PhD in Neurosciences, still at the same Faculty of Medicine, where I was given carte blanche to come up with a project. This project, my research baby, ended up with the title “Astrosomes: a novel approach for the regulation of neuronal communication” and consisted of the development of artificial cells that mimic astrocyte functions and can aid neurons against excitotoxicity.

After the PhD, still looking for new challenges, I moved to Sweden, where I am now a postdoctoral researcher in the Laboratory of Organic Electronics at Linköping University, studying plant electrophysiology and organic bioelectronics.

**Ave Bisesi (AB):** I’ve bounced around a fair bit in my academic career. My earliest science days were as an undergraduate at Oberlin College, where I studied biology and completed my thesis on mitogenomic evolution. After graduating, I worked for about a year as a research technician in a lab studying HIV viral evolution, pathogenesis, treatment and cure development, before returning to Oberlin to work in a position centered on increasing the retention of underrepresented minority students in STEM majors. Now, I’m a second-year PhD student in the Harcombe lab at the University of Minnesota in the Ecology, Evolution and Behavior program. My work is focused on the evolutionary ecology of microbial communities and the impact of bacteriophage (viruses that infect bacteria) on bacterial interactions.

What has been your proudest accomplishment so far?

**AAM:** Overall, I have to say that my proudest accomplishment so far has to be the successful execution of my PhD project. Back in 2016, when I first pitched it, I had no knowledge about synthetic biology or cell mimicry and was told that this project would be a very risky endeavor. Four years later, we were able to develop two generations of artificial cells and expand the project for in vivo testing and the development of artificial cells with different active components.

**AB:** As is the case for many academics, I find it difficult to identify an accomplishment of which I’m proudest - there’s always more work to do! - but getting accepted into PhD programs stands out, especially since it happened right at the beginning of the pandemic and was a source of hope in those initial days of uncertainty. The process of applying and interviewing is long and grueling, so it meant a lot that it had paid off and I could start to envision my next step.

Who has been a role model or key mentor in STEM (LGBTQIA + or otherwise) that has had an impact on your career?

**AAM:** I think role models can be divided into two categories. First, prominent scientists that paved the way for minorities in STEM. In this case, I would like to acknowledge Ben Barres, the first transgender neuroscientist that I encountered. His life story is inspiring and his research tremendously relevant for my field of study. Furthermore, I was lucky enough to attend a memorial event for him at the Society for Neuroscience conference and got to witness the legacy and affection that Ben left on everyone that crossed their paths with him.

On a second definition of role models, I consider people in my life who were pivotal in my career. Here, I would point out Dr. Constança Coelho, professor of Genetics at the Faculty of Medicine, who advised me to study biochemistry and that I always admired while growing up. I also consider my PhD supervisor, Dr. Sandra Vaz, to be a huge role model for me. She was as perfect as a supervisor can be, allowed me to be bold with my science, and showed me what a loving and supportive research environment can be.

**AB:** I’ve been fortunate to find wonderful, caring mentors at every stage of my career in biology - it’s the main reason I continued in the field. In fact, as an undergraduate, I’d originally wanted to be a math major, but after a few bad experiences I ended up switching to biology, because everyone I met through the department was so lovely and warm. Specifically, I worked under both Dr. Aaron Goldman and Dr. Angela Roles at various points. When I was still coming to terms with being trans and what exactly that meant for me, Dr. Goldman’s genomics class was the first time I remember hearing a biology professor explicitly grapple with the shortcomings of biological notions of sex and gender. It was affirming to see that process and realize that this field of study that I loved, a place where I’d found a sense of belonging, wasn’t fundamentally at odds with my lived experiences. As a research advisor and colleague, Dr. Roles has always been so supportive of me, my ideas and my goals, as she is of all her students. On top of doing brilliant science, she’s also an amazing example of what it looks like to show up in academia as your authentic self, and I’ve looked to her for that example many times in my career.

How do you think STEM could better promote inclusive research environments and support transgender scientists?

**AAM:** I think the most pressing issue in STEM regarding transgender scientists is the struggle to change our names in previous publications. This has affected me a lot. While some journals are moving with the times and understand the necessity of erasing a “dead name”, others simply offer an article correction - still maintaining the “dead name” on the article. This means that disclosing my transness is not my decision and never will be. The consequences of this are not only that I have something extra to explain in job interviews or need to supply extra documents for grants in order to prove that my research is my own. A more severe consequence is the potential for this to invite transphobia into my life. Considering that violence against transgender people is still a sad widespread reality, this refusal to protect transgender scientists can lead to tragic ends. Given this, there should be more awareness in STEM about the struggles that are specific to transgender scientists, in order to create a bigger support network.

**AB:** Departments and institutions should offer health insurance plans that provide coverage for gender-affirming care. For PhD students in particular, low stipends can be a barrier for trans people who are saving for expensive gender-affirming surgeries or who may not have any familial financial support. With the exception of instances in which a legal name is absolutely required, institutions should make it straightforward to designate and use a preferred name system-wide. Beyond that, I really believe that a lot of the work of inclusivity is interpersonal and proactive. Every lab and department should take the time to stop and consider what changes need to be made to the way they do things in order for a trans person to feel comfortable. You might not get everything right, but it’s obvious when institutions are being reactive rather than trying to design processes and spaces that are inclusive in the first place. But, most importantly, I can’t speak for all trans people. Your trans colleague knows what would best serve them, so listen. Make it clear that you will advocate for them, and then do so.

What advice would you give to other academic trainees struggling with their identity?

**AAM:** My advice is to reach out and create a network of people who followed the same path. Pursuing an academic career while discovering yourself at the same time can be an exhausting and isolating experience. While you may feel that no one around you understands what you are going through, some of us older folks do, and most of us would be honored to mentor you or just talk and be a friend. I know I would.

**AB:** This isn’t advice, per se, but I’d say that you belong. Whether that means staying in research or going elsewhere, you are meant to be there, you deserve to be there, and you make it better by being a part of it. Wherever you end up, you deserve colleagues that recognize your value and contributions, and you’re certainly not asking for too much by holding people accountable to using your pronouns or your name, etc. I’d also say, from experience, there’s no one right way to be a scientist and no one right way to be queer or trans. I’m certainly making it all up as I go along. Personally, one of the joys of transness has always been looking around at the world and asking why things are the way they are and if they can be different. Science, in my mind, revolves around those questions, too. Maybe, for you, queerness or transness or science is totally different. That’s the beauty of it. I celebrate that, and I celebrate you.

*Interviews were conducted by Associate Editor George Inglis*.

